# Nitrogen addition decreases methane uptake caused by methanotroph and methanogen imbalances in a Moso bamboo forest

**DOI:** 10.1038/s41598-021-84422-3

**Published:** 2021-03-10

**Authors:** Quan Li, Changhui Peng, Junbo Zhang, Yongfu Li, Xinzhang Song

**Affiliations:** 1 Center for Ecological Forecasting and Global Change, College of Forestry, Northwest A&F University, Yangling, 712100 China; 2 State Key Laboratory of Subtropical Silviculture, Zhejiang A&F University, Hangzhou, 311300 China; 3 Department of Biology Sciences, Institute of Environment Sciences, University of Quebec at Montreal, Case Postale 8888, Succursale Centre-Ville, Montreal, H3C3P8 Canada

**Keywords:** Tropical ecology, Ecology, Climate-change ecology, Forest ecology, Microbial ecology

## Abstract

Forest soils play an important role in controlling global warming by reducing atmospheric methane (CH_4_) concentrations. However, little attention has been paid to how nitrogen (N) deposition may alter microorganism communities that are related to the CH_4_ cycle or CH_4_ oxidation in subtropical forest soils. We investigated the effects of N addition (0, 30, 60, or 90 kg N ha^−1^ yr^−1^) on soil CH_4_ flux and methanotroph and methanogen abundance, diversity, and community structure in a Moso bamboo (*Phyllostachys edulis*) forest in subtropical China. N addition significantly increased methanogen abundance but reduced both methanotroph and methanogen diversity. Methanotroph and methanogen community structures under the N deposition treatments were significantly different from those of the control. In N deposition treatments, the relative abundance of *Methanoculleus* was significantly lower than that in the control. Soil pH was the key factor regulating the changes in methanotroph and methanogen diversity and community structure. The CH_4_ emission rate increased with N addition and was negatively correlated with both methanotroph and methanogen diversity but positively correlated with methanogen abundance. Overall, our results suggested that N deposition can suppress CH_4_ uptake by altering methanotroph and methanogen abundance, diversity, and community structure in subtropical Moso bamboo forest soils.

Methane (CH_4_) is the second-most important anthropogenic greenhouse gas after carbon dioxide^1^ and is responsible for 15% of the effects of global warming^2^. The atmospheric concentration of CH_4_ has been increasing rapidly in the past decades^1,3,4^ owing to a growing imbalance between production and consumption^5^. In soils, CH_4_ is mainly produced by methanogens during organic decomposition^6^; in aerobic soils, it is then consumed via oxidation by methanotrophs^7^, whereas in anaerobic conditions, it is consumed by anaerobic methanotrophs^8,9^, such as sulfate-dependent, nitrate- or nitrite-dependent, and metal-dependent CH_4_ oxidizers^10^. Methanotrophs are classified into two groups (type-I and type-II) according to their phylogenetic affiliations, carbon assimilation pathways, and phospholipid fatty acid compositions^11^ and the terms (type-I and type-II) are frequently used and adapted to the increasing diversity of methanotrophs^12^. Numerous studies have demonstrated that changes in methanogens and methanotrophs activities depend on temperature, moisture, and nutrient availability^13–15^. In particular, nitrogen (N) can directly affect methanotrophs and methanogens at the cellular level or indirectly influence them by inducing changes in the soil ecosystem^16^.

The annual input of reactive anthropogenic N in soils has increased more than tenfold in the past 150 years, and this trend is predicted to intensify by 2- or threefold in the coming years^17–19^. The largest N increases are likely to continue occurring in both East and South Asia^20,21^, particularly in subtropical China^22,23^. The response of methanotrophs to N addition and the subsequent change in CH_4_ emission rates are inconsistent; multiple contradictory results have been published, including evidence of methanotroph inhibition^24,25^, stimulation^26,27^, and null responses^28^. Many studies have also highlighted the highly complex nature of the effects of N addition on methanogen activity in soils^7^. For example, Shang et al*.*^29^ demonstrated that the addition of urea stimulates methanogen activity in rice soils owing to an increase in biomass production. In contrast, the application of N fertilizer (100 and 300 kg N ha^−1^ yr^−1^) has been shown to decrease methanogen activity in rice soils^30^. Furthermore, the abundance, diversity, and community composition of methanotrophs and methanogens are key determinants of their ecological functions^31–33^. Some studies have found that N addition affects the abundance and community composition of methanotrophs and methanogens^15,34–36^. For example, Aronson et al.^15^ found that ammonium nitrate addition (67 kg NH_4_NO_3_ ha^−1^ yr^−1^) increases methanotroph and methanogen abundance and the richness of methanotrophs in poorly-drained pine forest soil but decreases the richness of methanotrophs and methanogens in well-drained pine forest soil. The application of N fertilizer (compound fertilizer + urea 20.8 kg N ha^−1^ yr^−1^) decreases the abundance of methanotrophs and methanogens and significantly affects type-I methanotrophs but does not affect methanogen community composition in orchard plots^35^. In addition, the effects of different N application rates and types (NH_4_^+^, NO_3_^−^, or urea) on methanotroph communities are different and may depend on the ecosystem type^37,38^. Zhang et al.^39^ found that NH_4_^+^ addition (45 kg N ha^−1^ yr^−1^) decreases the abundance of methanotrophs and affects methanotroph community composition in temperate forest soils. Mohanty et al.^40^ observed that the application of NH_4_NO_3_ (60 kg N ha^−1^ yr^−1^) increases the abundance of type-I methanotrophs but decreases that of type-II methanotrophs in incubated forest soils. In rice soil, the addition of NH_4_^+^ suppresses type-II but stimulates type-I methanotrophs, whereas the addition of NO_3_^−^ increases both types of methanotrophs^41^ and that of urea does not alter the methanotroph community^42^. Urea and ammonia addition significantly increase the diversity of methanotrophs, whereas NO_3_^−^ addition only favors type-I methanotrophs in an alpine marsh meadow in the Qinghai-Tibetan plateau^38^. Jang et al.^43^ found that NH_4_NO_3_ addition inhibits type-I methanotrophs in temperate forest soils. Most of these studies either only observed methanotrophs^39,43^, were performed in manipulation experiments^40^, or were carried out in temperate forest soils to assess the effects of N addition on methanotrophs and methanogens^15^. Therefore, more information regarding different forest soils, especially tropical or subtropical forest soils, is necessary to advance our understanding of methanotroph and methanogen dynamics under conditions of increasing N deposition.

In China, there are 4.43 million hectares of Moso bamboo (*Phyllostachys edulis*) forest, which comprise 70% of the total bamboo forest area and 2% of the total forest area in the country^44^. Moso bamboo can grow to a height of 10–20 m in 40–50 days^45^. In subtropical China, the mean annual bulk N deposition has reached 30 kg N ha^−1^ yr^−1^^46^ and is predicted to remain high for the foreseeable future^20,47^. Several field studies have observed that simulated N deposition suppresses CH_4_ uptake in tropical forests in southern China, which may be attributed to the increase in inorganic N, soil Al^3+^ release, and the drop in pH owing to N addition^48,49^. In addition, our previous study in the same site found that N addition significantly reduces soil CH_4_ uptake in Moso bamboo forests, which is attributed to abiotic factors, such as the change in soil NH_4_^+^ concentration and pH^50^. Previous studies have demonstrated that the abundance, diversity, and community structure of methanotrophs and methanogens are mainly influenced by soil pH^51,52^, NH_4_^+^ concentration^16^, and soil substrate^53^. For example, pH has a negative effect on upland soil cluster (USC)-α and a positive effect on USC-γ abundance^54^. USC-α has been detected in mostly acidic upland soils^55^, whereas USC-γ is detected in alkaline upland soils^56,57^. Furthermore, few studies have linked CH_4_ fluxes to the abundances of methanotrophs and methanogens and the environmental factors influencing their abundances^54^. Thus, it is equally important to investigate N deposition on the relationship between soil CH_4_ flux and the abundance and community structure of the methanotrophs and methanogens in Moso bamboo forests.

The *pmoA* gene, which is commonly recognized as a phylogenetic marker of methanotrophs in ecological studies, encodes the membrane-bound subunit of particulate methane monooxygenase (MMO)^58^. MMOs catalyze the conversion of NH_4_^+^ and dioxygen to methanol and water, with one atom of the dioxygen molecule being incorporated into methanol and the other into water^59^. The *mcrA* gene encodes the alpha subunit of methyl coenzyme M reductase (MCR), which is the key catabolic enzyme of methanogens^60^; it catalyzes the reduction of a methyl group bound to coenzyme M, releasing CH_4_^61^. Therefore, it is widely accepted that the abundance and community composition of methanotrophs and methanogens are characterized by the *mcrA* and *pmoA* genes, respectively. Here, we investigated how N deposition affects methanotroph and methanogen abundance, diversity, and community structure in a Moso bamboo forest using the *pmoA* and *mcrA* genes. This study tested the following hypotheses: (1) N addition will decrease methanotroph abundance and influence methanotroph community structure and diversity; (2) N addition will decrease methanogen abundance and influence methanogen community structure and diversity; and (3) N addition will inhibit CH_4_ uptake by altering methanotroph and methanogen abundance, diversity, and community structure. The information is important to our understanding of how increasing N deposition could change the abundance, community structure, and diversity of soil methanotroph and methanogen and the methane flux they drive in the Moso bamboo plantations in the future.

## Results

### Soil properties

N deposition has an important impact on soil physicochemical properties (Table 1). The highest soil pH (4.9) was recorded from the control treatment; it was significantly higher than that in the other treatments (*P* < 0.05), especially compared with the N90 treatment (pH 4.2). Concentrations of NO_3_^−^ and NH_4_^+^ and the C/N ratio were higher in the N90 treatment than in the control treatment (*P* < 0.05). Compared with the control treatment, the higher amount of N addition (N90) significantly decreased moisture and the concentrations of soil organic carbon (SOC) and total nitrogen (TN). The concentration of available phosphorus (AP) increased after N addition compared with the control treatment (*P* < 0.05).

**Table 1:** Average physicochemical properties of the soil in the Moso bamboo forest study plots.

Properties	Control	N30	N60	N90
Moisture (%)	34.2 ± 1.9a	32.3 ± 2.7b	35.9 ± 2.9a	32.0 ± 3.6b
pH	4.9 ± 0.07a	4.4 ± 0.03c	4.5 ± 0.01b	4.2 ± 0.03d
SOC (g kg^−1^)	26.2 ± 2.6a	22.7 ± 1.9c	25.6 ± 3.7ab	24.9 ± 2.3b
MBC (mg kg^−1^)	637.1 ± 62.7b	574.3 ± 14.7b	803.3 ± 74.7a	718.3 ± 27.8ab
DOC (mg kg^−1^)	234.0 ± 17.1ab	251.3 ± 17.5a	215.7 ± 6.6b	206.1 ± 13.2b
TN (g kg^−1^)	2.2 ± 0.02a	1.8 ± 0.03c	2.1 ± 0.02b	1.9 ± 0.03c
NO_3_^-^ (mg kg^−1^)	16.1 ± 1.5c	12.8 ± 1.2d	24.7 ± 2.5a	18.2 ± 1.7b
NH_4_^+^ (mg kg^−1^)	10.9 ± 1.3b	11.3 ± 1.7b	7.0 ± 0.5c	18.9 ± 2.4a
C/N	11.7 ± 1.3b	12.3 ± 0.7b	11.8 ± 1.1b	13.4 ± 0.9a
AP (mg kg^−1^)	7.3 ± 0.9d	32.0 ± 1.2a	20.1 ± 1.6b	14.6 ± 1.1c

*SOC* soil organic carbon, *MBC* microbial biomass carbon, *DOC* dissolved organic carbon, *TN* total nitrogen, NO_3_^−^ nitrate, NH_4_^+^ ammonium, *C/N* carbon/nitrogen, *AP* available phosphorous.

Mean ± SD (n = 3). Different lower-case letters indicate significant differences at *P* < 0.05 for all treatments.

### Methanotroph and methanogen abundance

**Figure 1:**
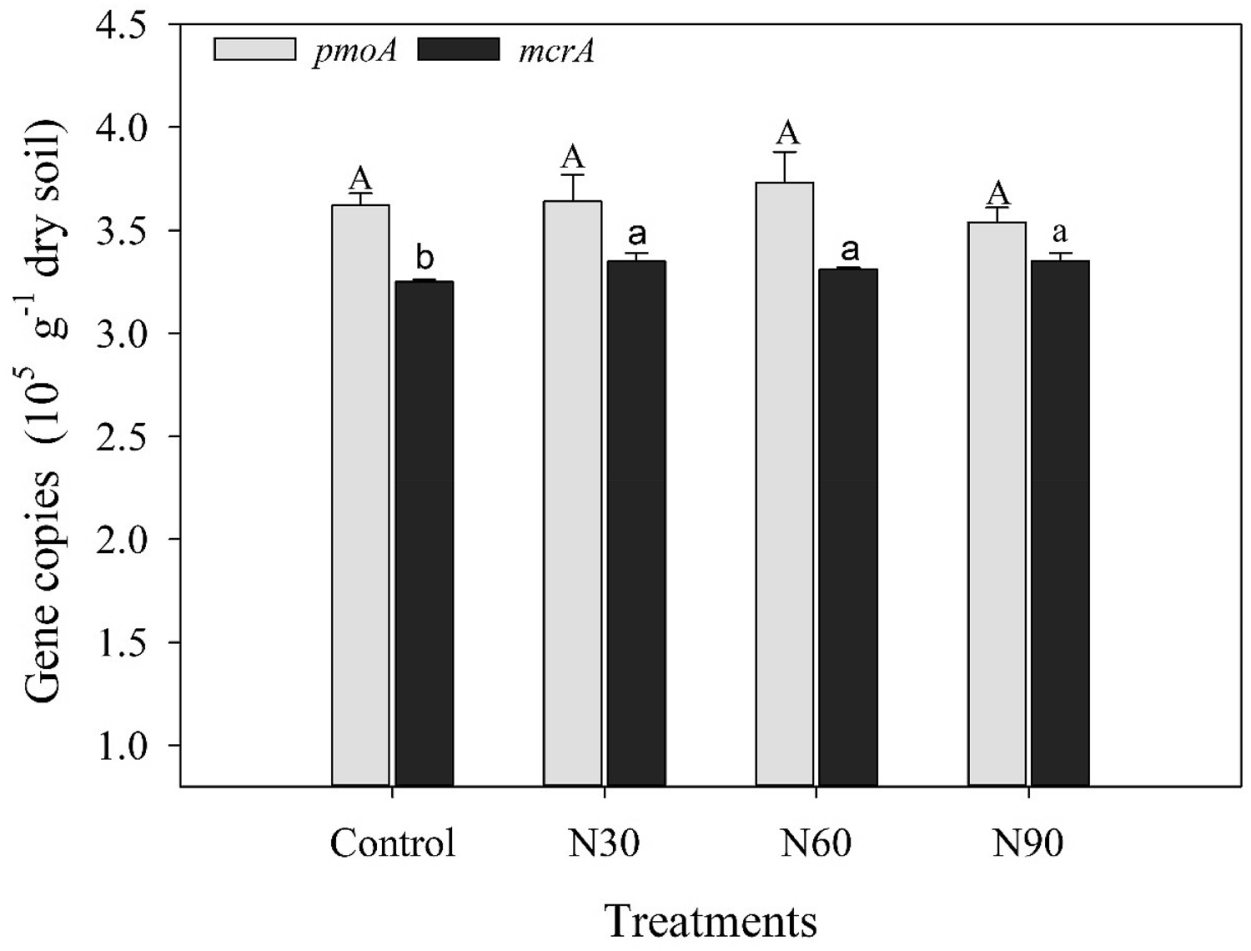
Methanotroph (*pmoA*) and methanogen (*mcrA*) abundance under different N addition treatments in Moso bamboo forest soil (Control, 0 kg N ha^−1^ yr^−1^; N30, 30 kg N ha^−1^ yr^−1^; N60, 60 kg N ha^−1^ yr^−1^; N90, 90 kg N ha^−1^ yr^−1^). Different upper-case letters indicate significant differences (*P* < 0.05) between treatments for methanotrophs and different lower-case letters indicate significant differences (*P* < 0.05) between treatments for methanogens.

**Figure 2:**
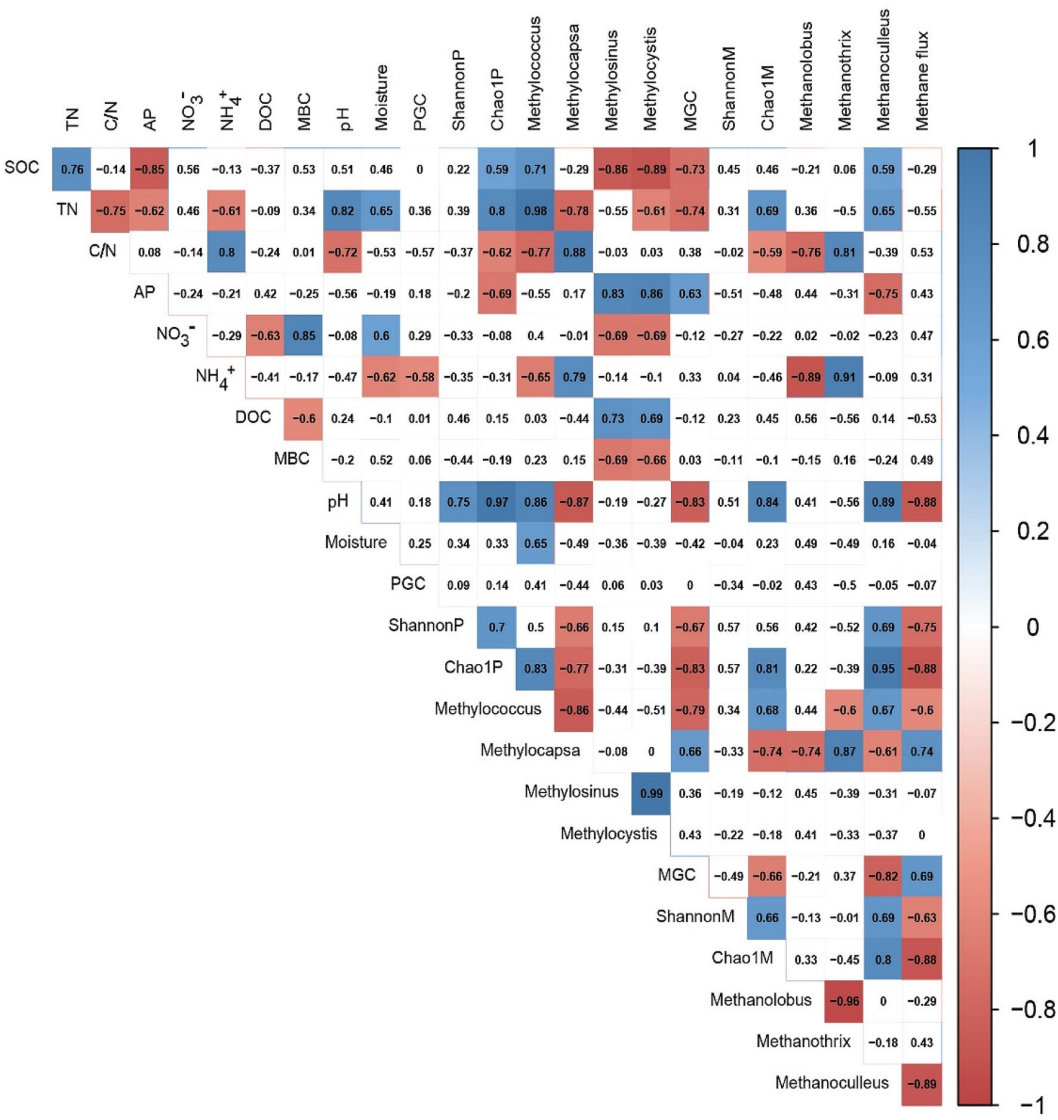
Pearson’s correlation coefficients (R) for relationships between soil properties and methanotroph and methanogen abundance, diversity, and dominant community, and CH_4_ flux. SOC, soil organic carbon; MBC, microbial biomass carbon; DOC, dissolved organic carbon; TN, total nitrogen; NO_3_^-^, nitrate; NH_4_^+^, ammonium; C/N, carbon/nitrogen; AP, available phosphorous; PGC, abundance of *pmoA* gene; ShannonP, Shannon index for methanotrophs; Chao1P, Chao1 index for methanotrophs; MGC, abundance of *mcrA* gene; ShannonM, Shannon index for methanogens; Chao1M, Chao1 index for methanogens. The color and numbers shown indicate the strength and sign of the correlation. Lack of color indicates no significant correlations (*P* > 0.05). Cool colors indicate significant and positive correlations (*P* < 0.05), whereas warm colors indicate significant and negative correlations (*P* < 0.05).

N addition did not significantly affect the abundance of the *pmoA* gene (*P* > 0.05; Fig. 1). The abundance of the *mcrA* gene in the control treatment was significantly lower than that in the N addition treatments (*P* < 0.05; Fig. 1). The correlation analysis revealed that the abundance of the *pmoA* gene was negatively correlated with the soil NH_4_^+^ concentration (*P* < 0.05). The abundance of the *mcrA* gene was positively correlated with the AP concentration (*P* < 0.05) but negatively correlated with soil SOC and TN concentrations and pH (*P* < 0.05; Fig. 2).

### Methanotroph and methanogen diversity

**Figure 3:**
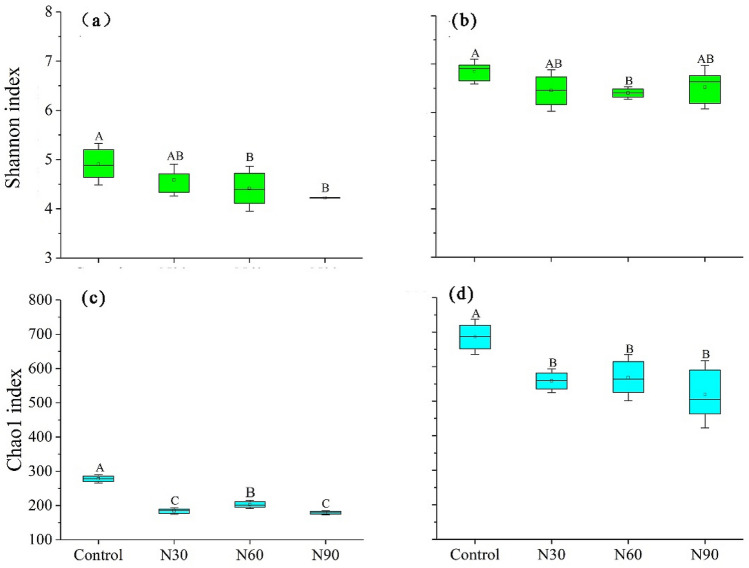
Shannon and Chao1 indexes for methanotrophs (**a**, **c**) and methanogens (**b**, **d**) under different N addition treatments. Different letters indicate significant differences (*P* < 0.05) between treatments.

The Shannon and Chao1 indexes were used to estimate soil microbial diversity and richness among all treatments (Fig. 3). The values of the Shannon and Chao1 indexes for methanotrophs were significantly lower in the N addition treatments than in the control treatment (*P* < 0.05). The Chao1 index for methanogens decreased significantly after N addition in all treatments (*P* < 0.05), whereas the Shannon index only decreased significantly in the N60 treatment (*P* < 0.05). The Shannon and Chao1 indexes for methanotrophs were positively correlated with soil pH (*P* < 0.05). In addition, the Chao1 index for methanotrophs was negatively correlated with AP concentration (*P* < 0.05) and the C/N ratio (*P* < 0.05) but positively correlated with SOC (*P* < 0.05) and TN (*P* < 0.05; Fig. 2) concentration. Pearson’s correlation analysis revealed that the Chao1 index for methanogens was positively correlated with soil pH (*P* < 0.05) and TN concentration (*P* < 0.05) but negatively correlated with the C/N ratio (*P* < 0.05).

### Methanotroph and methanogen community structure

**Figure 4:**
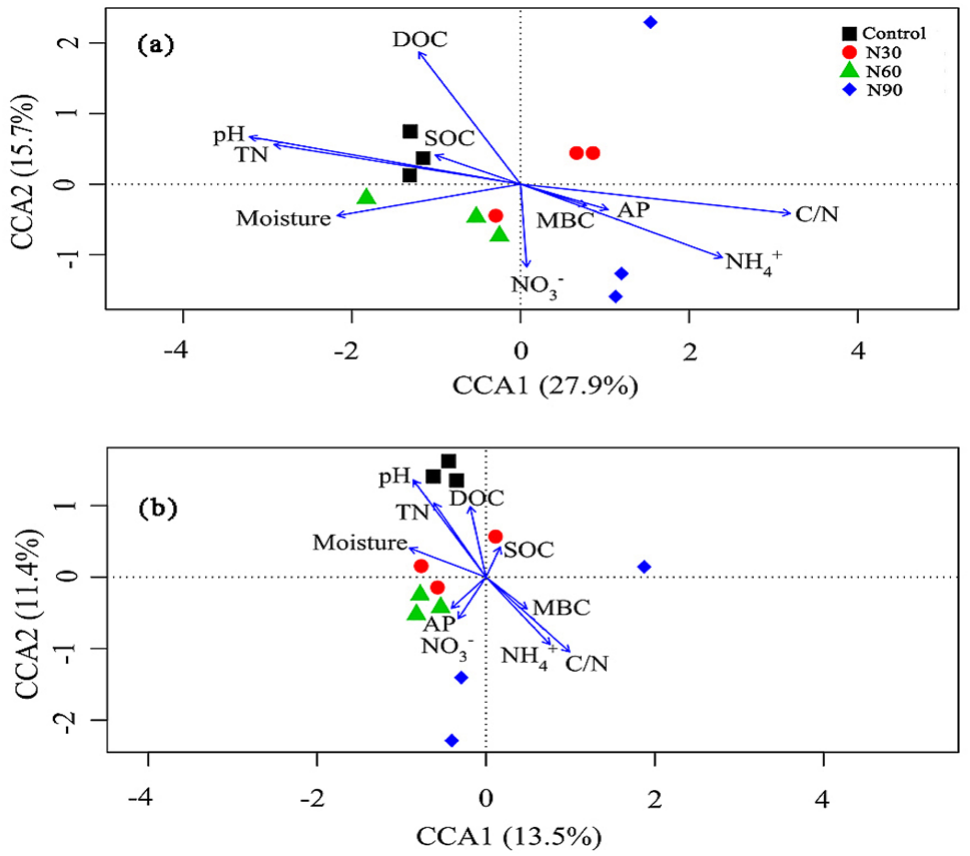
Results of the canonical correlation analysis (CCA) of the relationships between soil physicochemical properties and methanotroph (**a**) and methanogen (**b**) community structure in all treatments.

The number of operational taxonomic units (OTUs) detected varied across the N addition treatments (Fig. S1). The *pmoA* OTUs ranged from 231 in the N90 treatment to 349 in the control treatment. For *mcrA*, a total of 1050 OTUs were detected in the control treatment; in the N30, N60, and N90 treatments, 898, 867, and 1157 OTUs were detected, respectively. When the four treatments were compared, we found that they shared 231 OTUs. Cluster analysis showed that the methanotroph and methanogen community structure in the control treatment was different from the structure observed in the N deposition treatments (Fig. S2). In addition, ANOSIM showed that there were significant differences between control and N addition treatments for the methanotroph (R = 0.75, *P* < 0.001) and methanogen (R = 0.58, *P* < 0.001) community structure (Table S1). The canonical correspondence analysis showed that soil characteristics were related to the methanotroph and methanogen community structure (Fig. 4). Furthermore, a Monte Carlo permutation test showed that soil pH, the C/N ratio, and TN and NH_4_^+^ concentration (*P* < 0.05) were the primary factors that influenced methanotroph communities (Table 2). For methanogens, soil pH and the C/N ratio were the two most important contributors to the variation in methanogen communities (*P* < 0.05; Table 2).

**Table 2:** Monte Carlo permutation test correlations between methanotroph and methanogen community structure and soil physicochemical properties.

Soil variables	Methanotroph		Methanogen	
	r^2^	*P*	r^2^	*P*
Moisture	0.385	0.142	0.287	0.252
pH	0.847*	0.002	0.786*	0.005
SOC	0.093	0.684	0.062	0.843
MBC	0.055	0.841	0.132	0.717
DOC	0.374	0.164	0.305	0.366
TN	0.696*	0.009	0.435	0.124
NO_3_^-^	0.1	0.698	0.134	0.705
NH_4_^+^	0.537*	0.041	0.437	0.202
C/N	0.816*	0.001	0.625*	0.037
AP	0.093	0.698	0.107	0.741

*Indicates significant effects (*P* < 0.05).

### Dominant methanotroph and methanogen groups

**Figure 5:**
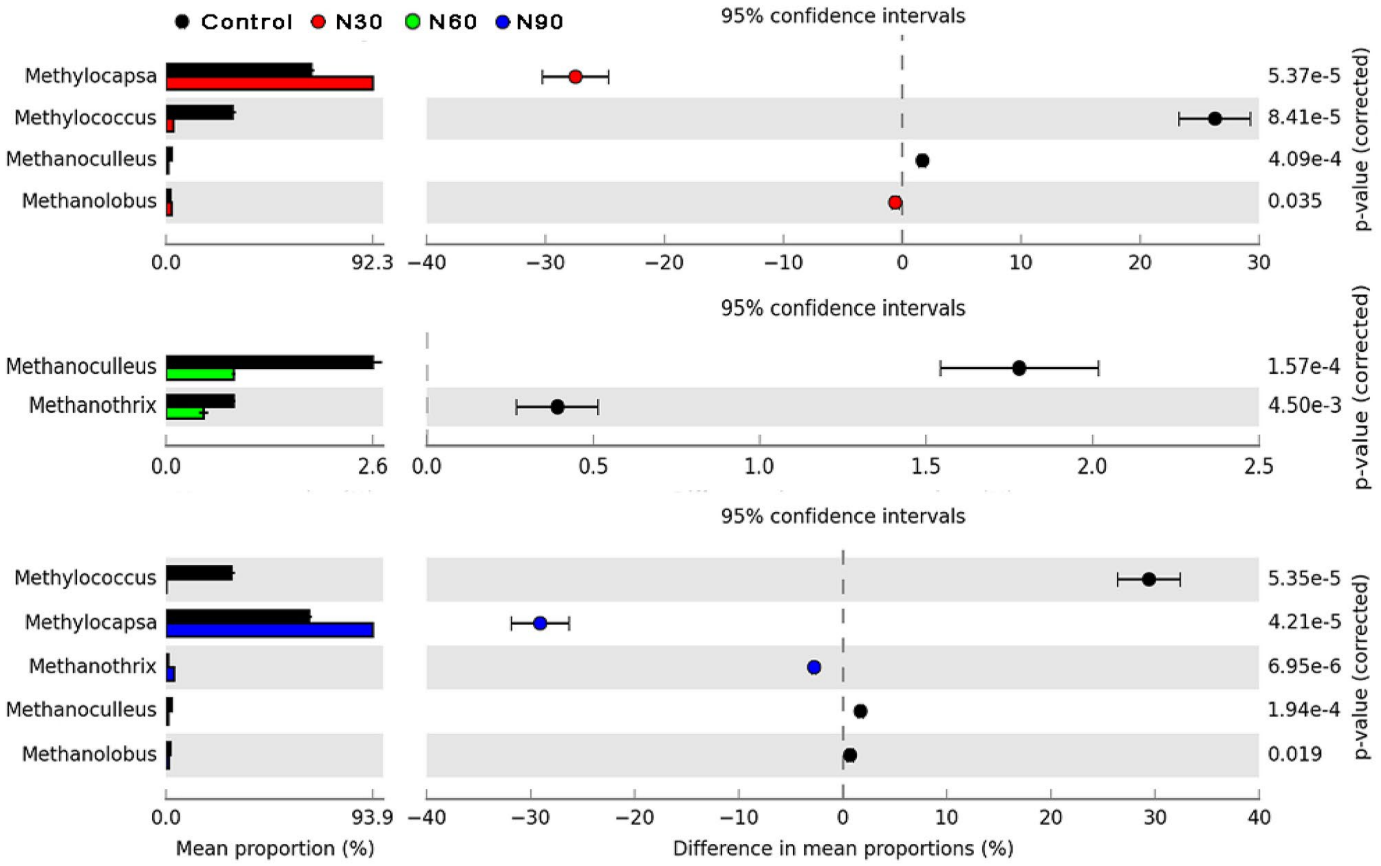
Dominant methanotrophs (*pmoA*) and methanogens (*mcrA*) under N addition treatments.

At the methanotroph genus level, four genera were most abundant (*Methylococcus*, *Methylocapsa*, *Methylosinus*, and *Methylocystis*) and presented relative abundances > 1% in all treatments (Fig. S2). *Methylocapsa* and *Methylococcus* were the two most abundant genera across all treatments and together accounted for 80.32–97.24% of the *pmoA* gene sequences. The relative abundance of *Methylocapsa* in the N addition treatments (N30 and N90) was significantly higher than that in the control treatment (Fig. 5), whereas *Methylococcus* showed the opposite trend. The relative abundance of *Methylocapsa* was negatively correlated with soil pH and TN concentration (*P* < 0.05) but positively correlated with the C/N ratio and NH_4_^+^ concentration (*P* < 0.05), whereas *Methylococcus* presented the opposite trend (*P* < 0.05; Fig. 2). The relative abundance of *Methylosinus* and *Methylocystis* was negatively correlated with SOC, microbial biomass carbon (MBC), and NO_3_^−^ concentrations (*P* < 0.05) but positively correlated with soil AP and dissolved organic carbon (DOC) concentrations (*P* < 0.05; Fig. 2).

For methanogens, *Methanolobus*, *Methanothrix*, and *Methanoculleus* (relative abundance > 1%) were the dominant genera across all sequence data (Fig. S2). The N addition treatments significantly decreased the relative abundance of *Methanoculleus* (Fig. 5). The relative abundance of *Methanothrix* was positively correlated with the C/N ratio and NH_4_^+^ (*P* < 0.05) concentration, whereas *Methanolobus* presented the opposite trend. *Methanoculleus* was positively correlated with soil pH (*P* < 0.05) and SOC (*P* < 0.05) and TN (*P* < 0.05) concentrations but negatively correlated with soil AP concentration (*P* < 0.05; Fig. 2).

### CH_4_ flux

**Figure 6:**
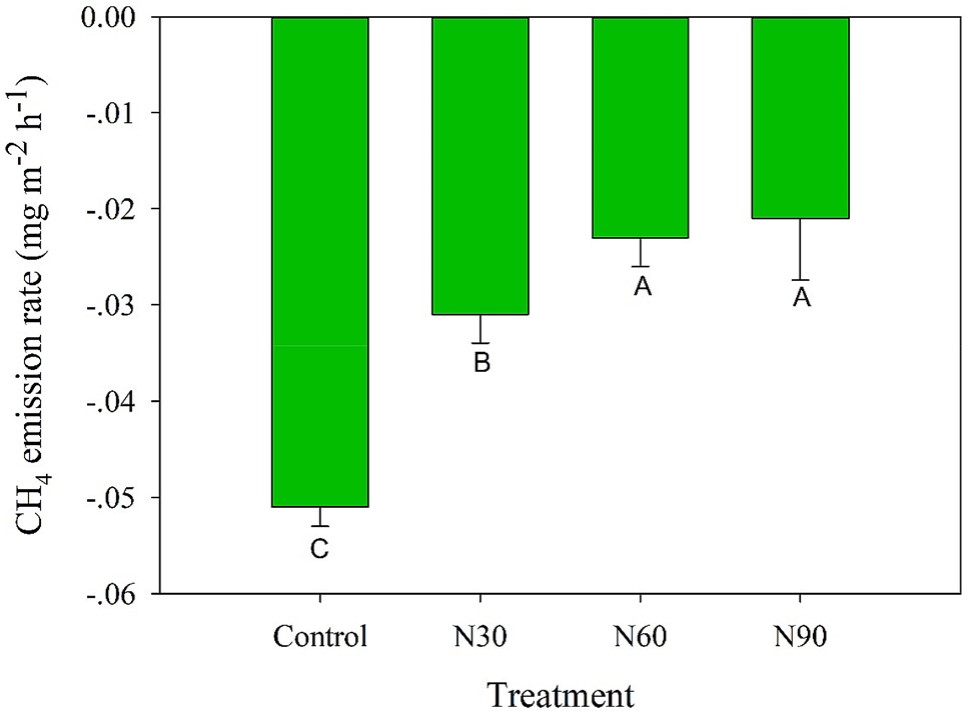
CH_4_ emission rate in February 2018 under different N addition treatments.

CH_4_ flux in the N addition treatments was significantly higher (39.2–58.8%) than that in the control treatment (Fig. 6). The CH_4_ flux was positively correlated with methanogen abundance and the relative abundance of *Methylocapsa* but negatively correlated with the Shannon and Chao1 indexes for both methanotrophs and methanogens, the relative abundance of *Methanoculleus* and *Methylococcus*, and pH (*P* < 0.05; Fig. 2).

## Discussion

### Effect of N addition on methanotroph abundance, diversity, and community structure

N addition did not significantly affect the abundance of the *pmoA* gene in the soil from the Moso bamboo forest; this did not support the first hypothesis that N addition would decrease methanotroph abundance. However, previous studies have found that N addition reduces the abundance of the *pmoA* gene in rice soils^34^ and temperate forest soils^39^. These reductions may be the result of high NH_4_^+^ concentrations reducing methanotroph activity through inhibition or competition for MMO^16^. Nitrite toxicity owing to the nitrification of NH_4_^+^ may also inhibit methanotroph activity^62^. In our study, the NH_4_^+^ soil concentration was significantly and negatively correlated with methanotroph abundance, which supports the aforementioned conclusion that a high NH_4_^+^ concentration inhibits methanotroph abundance. Low levels of N addition (N30 treatment) did not significantly affect soil NH_4_^+^ concentration in the present study, which indirectly indicates that N addition has no effect on *pmoA* abundance.

The Chao1 index presents species richness information and is sensitive to changes in rare species^63^, whereas the Shannon index accounts for both species abundance and evenness^64^. The effect of N addition on both the Shannon and Chao1 indexes was negative, suggesting an overall decline in soil methanotroph diversity. Although few studies have focused on methanotroph diversity in forest soils^15^, some studies have demonstrated that N addition significantly decreases soil microbial diversity^65–68^. A meta-analysis found that N addition decreases soil microbial (bacteria and fungi) diversity among different ecosystems^33^ owing to a decrease in soil pH^69,70^. Our results also found that soil pH was lower in the N addition treatments and positively correlated with methanotroph diversity, which supports the conclusion that N addition reduces methanotroph diversity. The underlying mechanism may be that soil pH influences the growth of some microbial functional groups^71^. Low pH leads to the leaching of magnesium and calcium and the mobilization of aluminum^72^. When this occurs, some microbes may suffer magnesium- or calcium-limitation or aluminum toxicity, which result in decreased microbial diversity^33,66^.

N addition significantly influenced the methanotroph community structure and the relative abundance of type-I and type-II methanotrophs. This result supports the first hypothesis of this study and is consistent with the findings of Zhang et al.^39^ and Jang et al.^43^, who found that N addition significantly affects methanotroph community structure. These studies demonstrated that N addition affects the community structure of soil microbes by changing the inorganic N concentration, the C/N ratio, and pH in soils^33,73,74^. In our study, the changes in soil TN and NH_4_^+^ concentrations, the C/N ratio, and pH owing to N addition influenced the methanotroph genera present and subsequently altered the composition of the microbial community. One possible explanation for this result is that soil microbial communities are directly influenced by soil pH given that most microbial taxa exhibit a relatively narrow pH tolerance for growth^69,75^. For example, a decrease in optimum growth of only 25% would lead to a population being rapidly outcompeted by other microbial populations that were not growth-impeded^69^. These narrow pH optima for microbes would explain the strong relationship between microbial community composition and soil pH. Furthermore, previous studies found that different methanotrophs have different pH optima^11,51^. The C/N ratio also plays an important role in the regulation of microbial community structure^76^, which may be attributed to microorganisms using substrates with different C/N ratios^77^.

In the present study, we found that the soil methanotroph community was dominated by type-II methanotrophs (*Methylocapsa*, *Methylosinus*, and *Methylocystis*) in all treatments. In particular, *Methylocapsa* was the most abundant indicator of methanotroph species and accounted for 77.5% of all sequences. Previous studies have reported that type-II methanotrophs are the predominant group in forest soils^43,78^, which may be the result of the abundance of type-I and type-II methanotrophs being affected by the concentration of CH_4_^79^. Type-II methanotrophs have been found to dominate under low CH_4_ concentrations, whereas type-I methanotrophs have been found to dominate under high CH_4_ concentrations^43^. Bender and Conrad^80^ demonstrated that forest soils are exposed to low CH_4_ concentrations. Therefore, type-II methanotrophs are the predominant group in Moso bamboo forest soils. In addition, some studies have shown that *Methylocapsa* is a member of USC-α in forest soils with an acidic pH^81,82^. In this study, the relative abundance of *Methylocapsa* was significantly and negatively correlated with soil pH (*P* < 0.01), which was consistent with the finding of Täumer et al.^38^ that there is a negative correlation between the pH and USC-α. However, the relative abundance of *Methylococcus* was strongly and positively correlated with soil pH (*P* < 0.01), which indicates that type-I methanotrophs were not able to adapt to the lower pH conditions of the soil in the N addition treatments. These results demonstrated that pH played an important role in altering the community composition of soil methanotrophs. Overall, the effects of N addition on methanotroph community structures in Moso bamboo forest soils were consistent with the results from temperate forest soils^39,43^. These results indicate that the response of methanotroph community structures to N addition in a subtropical forest ecosystem is similar to that in different forest ecosystems.

### Effect of N addition on methanogen abundance, diversity, and community structure

N addition significantly increased the abundance of the *mcrA* gene but decreased methanogen diversity, which partly supports the second hypothesis. Aronson et al.^15^ observed that the abundance of the *mcrA* gene is greater with N treatments than with control treatment in a pine forest soil. High N concentrations stimulate multiple microbial processes and provide more substrate for methanogens compared with low N concentrations^53^. DOC could partly act as the substrate and affect soil microbial activity^83^. In this study, N addition (N30 treatment) promoted an increase in the DOC concentration, which could explain the increase in methanogen abundance under conditions of N addition. Furthermore, our previous studies found that N addition increases the leaf photosynthetic rate^84^, soil MBC^66^, soil respiration rate^23^, and the decomposition rates of both leaf litter^47^ and fine roots^65^, which indirectly supports the aforementioned conclusion. Our study also found that the soil SOC and TN concentrations were lower in the N addition treatments than in the control treatment and were negatively correlated with methanogen abundance, which partly supports the idea that N addition significantly increases *mcrA* gene abundance. Moreover, the Shannon and Chao1 indexes for methanogens sharply declined with decreasing soil pH (from pH 4.9 to 4.2) in the N addition treatments, which is likely owing, in part, to a small fraction of methanogens not being able to survive in low-pH soil. For example, the relative abundance of *Methanoculleus* was lower in the N addition treatments and was positively correlated with both the pH and the Chao1 and Shannon indexes for methanogens (Fig. 2). This result supports the conclusion that low pH resulted in a decrease in the relative abundance of some methanogens.

Methanogen community structure, like that of the methanotrophs, was influenced by N addition, which supports the second hypothesis of this study. Moreover, our results showed that soil pH was strongly correlated with methanogen community structure in Moso bamboo forest soils (*P* < 0.01). Some studies have found similar results^68,70^. For example, Lin et al.^68^ found that pH strongly controls microbial community structure in soils with N fertilization treatments. This result was attributed to most microbes having relatively narrow pH optima^69^. Our results also showed that soil pH was significantly and positively correlated with the relative abundance of *Methanoculleus* but negatively correlated with the amount of *mcrA* (Fig. 2), which supports the conclusion that soil pH plays a dominant role in determining the structure of methanogen communities. However, other soil physicochemical factors may also play important roles in determining soil microbial community patterns and cannot be ruled out. The soil C/N ratio also significantly influenced the methanogen community structure, which is consistent with the results of Wan et al.^85^, who found that the soil C/N ratio is the major determining factor of the structure of microbial communities in subtropical coniferous and broadleaf forest plantation soils. The soil C/N ratio can reflect the quality of the substrate for soil microorganism growth^85^. In general, microbial biomass and activity are constrained by the availability and quality of C and nutrients, which may shift the structure of microbial communities^86^. In fact, a few studies on methanogens have been performed in forest soils within the context of atmospheric N deposition^15,35^. Aronson et al.^15^ found that N addition increases methanogen abundance in the poorly drained pine forest soil but does not impact methanogen abundance in a well-drained site. In our study, we showed that N addition significantly influenced methanogen abundance, diversity, and community structure in Moso bamboo forest soils. The differences in these results may be attributed to the evaluation of different forest soil types, drainage conditions, and N addition rates. As such, it is important to study the effects of N addition on methanogens in a variety of forest soils.

### Effect of N addition on CH_4_ flux

The oxidation of CH_4_ from the atmosphere is an important function in forest ecosystems^5^. Our results indicate that N addition significantly decreased CH_4_ uptake in the Moso bamboo forest, which supports the third hypothesis of this study and is consistent with the results of previous studies that have shown a negative effect of N addition on CH_4_ oxidation in forest soils^18,87^. Mo et al.^48^ and Zhang et al.^49^ also observed that CH_4_ uptake in monsoon evergreen broadleaf forest soils is significantly reduced by N deposition in southern China. The decrease in CH_4_ uptake with N addition is probably owing to increased methanogen and decreased methanotroph abundances^7^. It has been found that the abundance of USC-α is positively correlated with CH_4_ uptake in forest soils^38^. Aronson and Helliker^87^ found that large amounts of available N inhibit methanotrophs in non-wetland soil systems. Similarly, we found that N addition decreased methanotroph diversity and altered the community structure of methanotrophs. Pearson’s correlation analysis demonstrated that methanotroph diversity (Shannon and Chao1 indexes) was strongly correlated with CH_4_ flux, which agrees with the findings of Schnyder et al.^32^, who deduced that the diversity of methanotrophic communities is important for CH_4_ oxidation. The result also provides direct evidence for the loss of microbial diversity with increasing N deposition rates, which results in altered ecosystem functions. Moreover, Shang et al.^29^ showed that methanogen activity is enhanced by N addition, which results in the production of more CH_4_. In our study, N addition significantly increased methanogen abundance, which was positively correlated with CH_4_ flux. Our results indicate that N deposition resulted in the suppression of CH_4_ uptake in Moso bamboo forest soils, thereby contributing to an increased concentration of atmospheric CH_4_. In addition, abiotic soil factors, such as pH, directly and indirectly influence CH_4_ flux^88^ by altering methanotroph and methanogen abundance, diversity, and community structure.

## Conclusions

The present study provides evidence that N deposition may influence methanotroph and methanogen abundance, diversity, and community structure by decreasing pH in Moso bamboo forest soil. Furthermore, N addition significantly decreased methanotroph and methanogen diversity, which may influence their ecosystem functions, such as CH_4_ uptake. Increasing the soil pH should be an effective intervention option to alleviate the effect of N deposition on methanotrophs and methanogens. In this study, we ignored the potential role of anaerobic methanotrophs and soil characteristics (horizon layering, hydrology, and oxygen availability) over soil depths. In a further study, we will investigate the effect of N addition on soil anaerobic methanotrophs and soil characteristics of different depths in the Moso bamboo plantation. Besides, the long-term effect of N deposition on methanotrophs and methanogens, the CH_4_ emission rate, and the associated underlying mechanisms should be evaluated in future studies.

## Materials and methods

### Experimental site and design

The field site was established in Qingshan Town, Hangzhou City (30° 14ʹ N, 119° 42ʹ E), Zhejiang Province, China. The soil type is classified as a ferrosol derived from granite^23,50^. Moso bamboo is an economically important bamboo species in Southeast China and the most important source of non-wood forest products in China^47^. The Moso bamboo forest at the study site was originally established in the late 1970s from a native evergreen broadleaf forest in sites of similar topography^65^. The Moso bamboo forest, with 11 understory herbal species, achieves a mean height of 0.1 m. Forest floor coverage is 5% with a total herbal biomass of 14.6 kg ha^−1^. The forest is influenced by a subtropical monsoon climate, with a mean annual temperature of 15.6 °C and mean annual precipitation of 1420 mm. The initial soil characteristics are summarized in Table S2.

Twelve (3 replicates per treatment × 4 treatments) randomly scattered plots (20 m × 20 m per plot) were established in November 2012. Adjacent plots were separated by a 20-m buffer zone. Four distinct N treatments were defined: Control (0 kg N ha^−1^ yr^−1^), N30 (30 kg N ha^−1^ yr^−1^), N60 (60 kg N ha^−1^ yr^−1^), and N90 (90 kg N ha^−1^ yr^−1^). The N-addition treatments were designed to simulate single (N30), double (N60), or triple (N90) ambient N deposition rates (30 kg N ha^−1^ yr^−1^) in the region^46^. NH_4_NO_3_ was used to simulate N deposition given that the N that is typically deposited through natural and anthropogenic processes is mainly in the form of NH_4_^+^ and NO_3_^−^^89,90^, which account for 56.1% and 43.9% of wet N deposition in China, respectively^91^. Different concentrations of NH_4_NO_3_ solution (mixed with 10 L of water) were sprayed over the plots each month starting from January 2013 to March 2018. Each control plot received 10 L of water.

### Soil sampling and physicochemical analysis

For each plot, bulk soil (0–20 cm depth) was collected in early March 2018 from ten randomly selected points and mixed to form one composite sample. The samples were transported to the laboratory in a constant temperature box (4 ℃) containing ice within hours of being collected. After visible stones, roots, and litter were removed using forceps, the soil samples were gently broken apart along natural-break points and thoroughly mixed. One portion of the soil sample was passed through a 2.0-mm sieve and stored at − 80 °C for subsequent DNA extraction, quantitative PCR, and high-throughput sequencing. Another portion of the soil was passed through a 2.0-mm sieve and subsequently divided into two parts for soil physicochemical property analysis. A part of each fresh sample was stored at 4 °C for the analysis of MBC, DOC, inorganic N (NH_4_^+^ and NO_3_^−^), and soil moisture. MBC was estimated using the chloroform fumigation-extraction method^92,93^. DOC was extracted with distilled water, passed through a 0.45-mm filter, and evaluated using a TOC analyzer (TOC-VCHP, Shimadzu, Kyoto, Japan). NH_4_^+^ and NO_3_^−^ were extracted with 2 M KCl and measured using a SmartChem 200 Discrete Analyzer (Alliance Instruments, Frepillon, France). Fresh soil samples were weighed and then dried in an oven at 105 °C to a constant weight to determine gravimetric soil moisture^94^. The other parts were air-dried and stored at room temperature (25 °C). Air-died soils were used to determine soil pH, SOC, TN, and AP. Soil pH was measured using a pH meter (FE20, Mettler-Toledo, Zurich, Switzerland) after a soil–water (1:2.5 dry w/v) mixture was created and shaken for 30 min. SOC and TN concentrations were measured using a Vario Max element analyzer (Elementar, Hanau, Germany). AP was extracted with 0.0125 M H_2_SO_4_ in 0.05 M HCl and its concentration was determined using the molybdenum blue method^84^.

### DNA extraction and quantitative PCR

Soil DNA was extracted from 0.3 g of soil after sampling using the Ezup Column Soil DNA Purification Kit (Sangon Biotech, Shanghai, China) according to the manufacturer’s protocol. The quality and concentration of the extracted DNA were evaluated by gel electrophoresis (0.8% agarose) and a NanoDrop spectrophotometer (NanoDrop Technologies, Wilmington, DE, USA), and the extracted DNA was subsequently stored at − 20 °C.

The primers A189f. (5ʹ-GGNGACTGGGACTTCTGG-3ʹ) and 650R (5ʹ-ACGTCCTTACCGAAGGT-3ʹ)^95^ and mlas-mod–F (5ʹ-GGYGGTGTMGGDTTCACMCARTA-3ʹ) and mcrA-rev-R (5ʹ-CGTTCATBGCGTAGTTVGGRTAGT-3ʹ)^96^ were used for *pmoA* and *mcrA* gene amplification, respectively. Functional methanotroph and methanogen genes were quantified using qPCR in a CFX connect Real-Time Detection System (Bio-Rad Laboratories Inc., Hercules, CA, USA). The DNA sample was used for qPCR after a tenfold dilution. There was a single dissolution curve peak. The qPCR reaction mixture contained 10 μL of 2 × ChamQ SYBR Color qPCR Master Mix, 2 μL of each primer (10 μM), 1 μL of DNA template (1–10 ng), and 7 μL of ddH_2_O. Amplification was initiated by denaturation at 95 °C for 3 min, followed by 35 cycles of denaturation at 95 °C for 20 s, annealing at 60 °C for 30 s, and extension at 72 °C for 20 s, and the plate was read at 80 °C. To generate a standard curve, individual clones with accurate inserts were cultured in Luria–Bertani medium and the plasmid DNA was extracted, purified, and quantified. Plasmid DNA was prepared in a tenfold dilution series to yield a standard curve covering six orders of magnitude (10^2^ to 10^8^ copies) per assay^35^. The qPCR assay was performed in triplicate for each replicate. The qPCR amplification average efficiencies were 97% and the R^2^ was 0.996.

### High-throughput sequencing and bioinformatics

PCR amplification was performed for each soil DNA extract, using the above-mentioned primers (A189f. and 650R and mlas-mod–F and mcrA-rev-R), in triplicate and combined into a single composite sample. This is because these primers are widely used to study upland soils and cover the most methanotrophs and methanogens^35,38,95,96^. The specificity of the primer, which had been checked by Primer-BLAST, was good. The amplicon size of *pmoA* and *mcrA* was 500 and 469 bp, respectively. The PCR products were subsequently purified with AMPure XT beads (Beckman Coulter Genomics, Danvers, MA, USA) and quantified by Qubit (Invitrogen Corporation, Carlsbad, CA, USA). The PCR amplicon pools were prepared for sequencing and library quality was assessed using an Agilent 2100 Bioanalyzer (Agilent, Palo Alto, CA, USA) and the Library Quantification Kit for Illumina (Kapa Biosciences, Woburn, MA, USA). Finally, high-throughput sequencing for *pmoA*/*mcrA* genes was carried out using a 2 × 300 bp paired-end Illumina MiSeq PE300 at LC-Bio Technology Co., Ltd, Hang Zhou, Zhejiang Province, China.

The obtained sequencing data were processed using the Quantitative Insights into Microbial Ecology (QIIME) pipeline^97^. Sequence data, including raw data and clean data, were filtered using Mothur. The proportion of chimeric sequences of *pmoA* and *mcrA* was 4.8% and 6.5%. The non-chimeric *pmoA* and *mcrA* gene reads were then checked for frameshift errors using the “FrameBot” tool^98–100^. The above analysis resulted in a total of 424,628 (ranging from 31,030 to 40,328 sequences per sample) and 775,842 (ranging from 36,438 to 40,328 sequences per sample) high-quality sequences of *pmoA* and *mcrA* in all samples, respectively (Table S3). To standardize the results, we resampled each sample using the sequence number of the sample with the least sequences and calculated the diversity indices based on this normalized data set^101,102^. The remaining high-quality sequences were clustered into OTUs at a 97% identity threshold using UCLUST. The taxonomic information of each OTU was annotated using the taxonomically determined reference sequences from the National Center for Biotechnology Information (NCBI v20180310) using BLAST^35,38,39^. The specific parameter settings of BLAST were as follows: the minimum identity was 70%, the minimum query coverage was 70%, the maximum E-value was 10^–5^, and the E-value interval multiple was 10 times. The detailed parameters and classification methods have been described by Liu et al.^35^. Alpha diversity was assessed by calculating the Chao1^63^and Shannon^64^ indexes in QIIME (Version 1.8.0). Furthermore, QIIME was used to calculate the weighted UniFrac, and unweighted pair group method with arithmetic mean clustering was conducted on the weighted UniFrac based on a previously published protocol^66^. All sequence data in this study are deposited in the Sequence Read Achieve database of NCBI under accession number SRP255341.

### CH_4_ measurement

CH_4_ samples were collected once each month on a clear day using a widely applicable static chamber and measured using gas chromatography techniques^103^. The sampling process has been described in a previous study^23^. In brief, the static chambers were made of opaque polyvinyl chloride panels, including a square base box (40 × 40 × 10 cm) and a U-shaped groove (50 mm wide and 50 mm deep) at the top edges that held a removable top (40 × 40 × 40 cm). In each plot, three boxes were installed 10 cm below the soil surface. The chamber tops were placed onto the base boxes during gas sampling, and the grooves were filled with water to act as an air seal. A small fan was installed inside the top of each chamber to generate turbulence during sampling. Sampling was conducted between 9:00 am and 11:00 am to minimize the influence of diurnal variation. Gas samples (60 mL) were extracted from the chamber at 0, 10, 20, and 30 min using polyurethane syringes and stored in gas sampling bags (Delin Ltd., Dalian, China). The CH_4_ concentrations were determined using a gas chromatograph (GC-2014; Shimadzu Corporation, Kyoto, Japan) within 1 day of sample collection. The CH_4_ flux was calculated using the following formula^103^:

1 $$F = \left( {\frac{dc}{{dt}}} \right) \times \left( {\frac{M}{{V_{0} }}} \right) \times \left( {\frac{273.15}{T}} \right) \times \left( \frac{V}{A} \right)$$

where *F* (mg m^−2^ h^−1^) is the soil CH_4_ flux; $\frac{dc}{{dt}}$ is the slope of the linear regression between the change in the CH_4_ concentration (*dc*) and the time (*dt*) in the chamber; *M* and *V*_0_ are the molar mass and molar volume of CH_4_ under standard conditions, respectively; T is the absolute air temperature during sampling; and V (m^3^) and A (m^2^) are the effective volume and bottom area of the chamber, respectively. Owing to the malfunction of the gas chromatograph in March 2018, the data of CH_4_ flux for that month were abnormal and, thus, were eliminated. The CH_4_ flux data collected in late February 2018, 12 days before the soil sampling in March, were used to analyze the correlation between CH_4_ flux and the abundance, diversity, and community structures of methanotrophs and methanogens in the present study.

### Statistical analysis

A one-way analysis of variance (ANOVA) was performed to assess the differences in the number of gene copies, Chao1 index, Shannon index, and CH_4_ flux among the different treatments. Post-hoc multiple comparisons were conducted using the least significant difference (LSD) method. All data were tested for homogeneity of variance and normality of distribution prior to conducting the ANOVA. The relative abundance of the major genera was analyzed using STAMP software (v2.1.3) with a correction for multiple comparisons using the Bonferroni method. Pearson’s correlation analysis was used to test the association among soil physicochemical variables, alpha diversity, the relative abundance of the major genera, and CH_4_ flux, across all treatments. All these analyses were performed using SPSS v. 18.0 (SPSS Inc., Chicago, IL, USA).

R software (Version 3.4.1) was utilized to conduct the following analyses. First, correlations between soil physicochemical variables and OTUs were calculated with the vegan package using a Monte Carlo permutation test, canonical correspondence analysis (CCA), and analysis of similarities (ANOSIM). Venn diagrams for graphical descriptions of unique and shared OTUs between different ecosystems were generated using the VennDiagram package.

## Supplementary Information

Supplementary Information
