# Supplementary material for: Nitrogen addition decreases methane uptake caused by methanotroph and methanogen imbalances in a Moso bamboo forest
Source: Sci Rep. 2021 Mar 10;11:5578. doi: 10.1038/s41598-021-84422-3 (PMC7947007; doi:10.1038/s41598-021-84422-3)
Supplement: Supplementary file 1 — Supplementary Information [file 41598_2021_84422_MOESM1_ESM.docx]

**Nitrogen deposition decreases methane uptake caused by methanotroph and** **methanogen imbalances in a Moso bamboo forest**

Quan Li^1,2^, Changhui Peng^1,3*^, Junbo Zhang^2^, Yongfu Li^2^, Xinzhang Song^2*^

*^1^Center for Ecological Forecasting and Global Change, College of Forestry, Northwest A&F University, Yangling, 712100, China*

*^2^State Key Laboratory of Subtropical Silviculture,* *Zhejiang A&F University, Hangzhou, 311300, China*

*^3^Institute of Environment Sciences, Department of Biology Sciences,* *University of Quebec at Montreal,* *Case Postale 8888, Succursale Centre-Ville, Montreal, H3C3P8,*

*Canada*

*** Corresponding authors:**

**Changhui Peng**

Email: peng.changhui@uqam.ca

Tel: 1-514-987-3000 ext. 3041

Fax: 1-514-987-4718

**Xinzhang Song**

Email: [songxinzhang@gmail.com](mailto:songxinzhang@gmail.com)

Tel: (86) 571-63741816

Fax: (86) 571-63740809

**Supplemental Materials**

**Table S1.** Analysis of similarities under all treatments for the methanotroph and methanogen

| Genera | R | P |
| --- | --- | --- |
| Methanotroph | 0.75 | 0.001 |
| Methanogen | 0.58 | 0.001 |

**Table S2.** Original surface soil (0–20 cm) characteristics of the study plots in the Moso bamboo forest (mean ± SD, n = 3)

|  | Control | N30 | N60 | N90 |
| --- | --- | --- | --- | --- |
| SBD（g cm^-3^） | 0.97±0.07 | 0.98±0.04 | 1.00±0.05 | 0.94±0.03 |
| SOC（g kg^-1^） | 23.73±0.24 | 22.56±1.17 | 23.15±1.74 | 25.34±2.16 |
| TN（g kg^-1^） | 1.11±0.04 | 1.17±0.09 | 1.21±0.12 | 1.13±0.06 |
| TP（g kg^-1^） | 0.52±0.01 | 0.57±0.05 | 0.54±0.03 | 0.56±0.03 |
| pH | 4.46±0.01 | 4.43±0.04 | 4.48±0.08 | 4.42±0.06 |

SBD, soil bulk density; SOM, soil organic carbon; TN, total N; TP, total P

**Table S3.**  Usable sequences across the experimental plots

| Sample | Control_1 | Control_2 | Control_3 | N30_1 | N30_2 | N30_3 | N60_1 | N60_2 | N60_3 | N90_1 | N90_2 | N90_3 | Total |
| --- | --- | --- | --- | --- | --- | --- | --- | --- | --- | --- | --- | --- | --- |
| *pmoA* | 34499 | 32088 | 40328 | 35572 | 31030 | 32194 | 33153 | 38555 | 36391 | 37382 | 35560 | 37876 | 424628 |
| *mcrA* | 36438 | 66279 | 76568 | 51789 | 74796 | 96082 | 51780 | 59330 | 63917 | 55656 | 58308 | 84899 | 775842 |

**Figure Legends**

**Figure S1.** Venn diagrams showing the OUT type distribution for methanotrophs and methanogens in different N deposition treatments (Control, 0 kg N ha^-1^ yr^-1^; N30, 30 kg N ha^-1^ yr^-1^; N60, 60 kg N ha^-1^ yr^-1^; N90, 90 kg N ha^-1^ yr^-1^) in the Moso bamboo forest.

**Figure S2.** Cluster analysis for different N deposition treatments based on soil methanotroph (a) and methanogen (b) communities in the Moso bamboo forest soil.


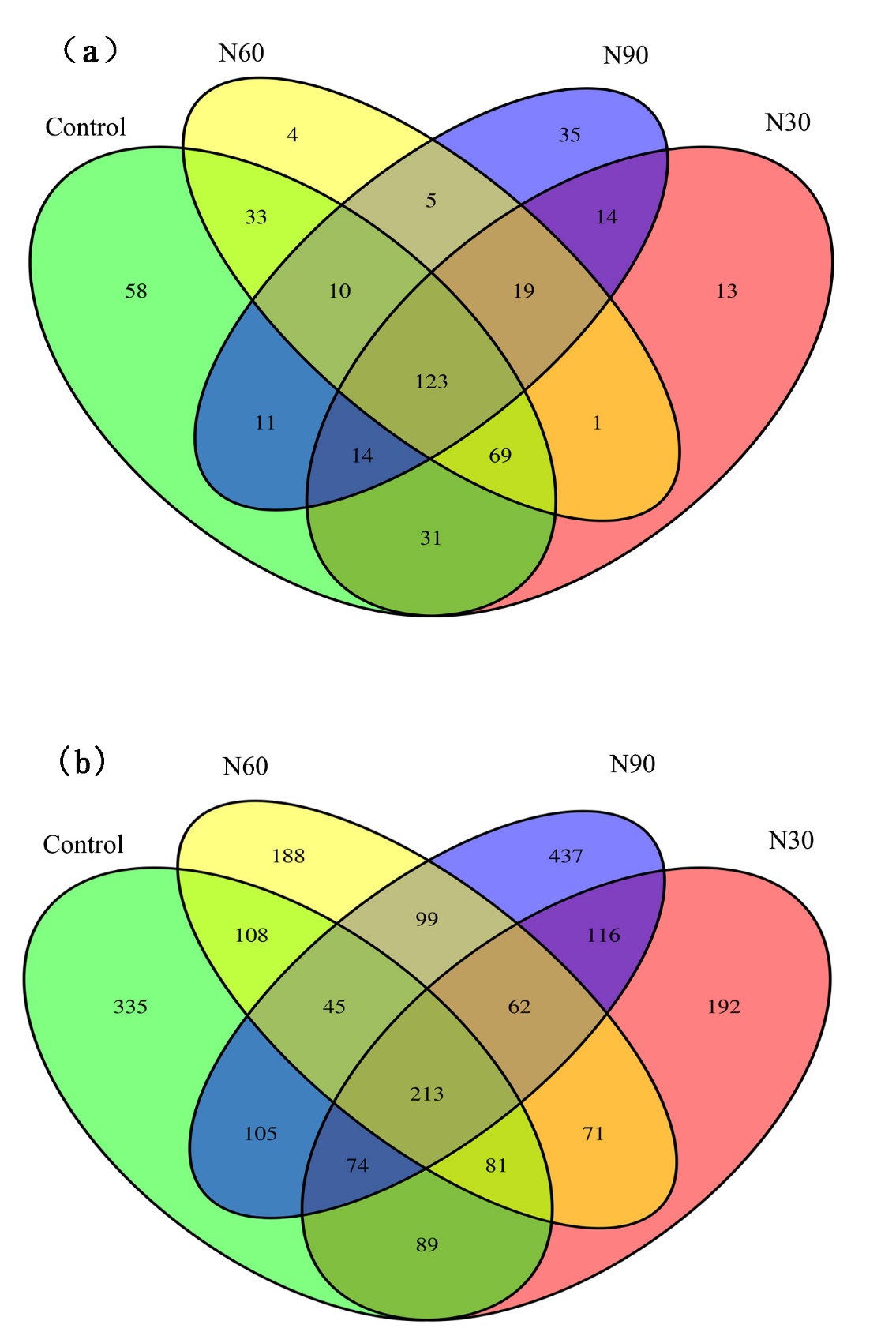


**Fig. S1**

**
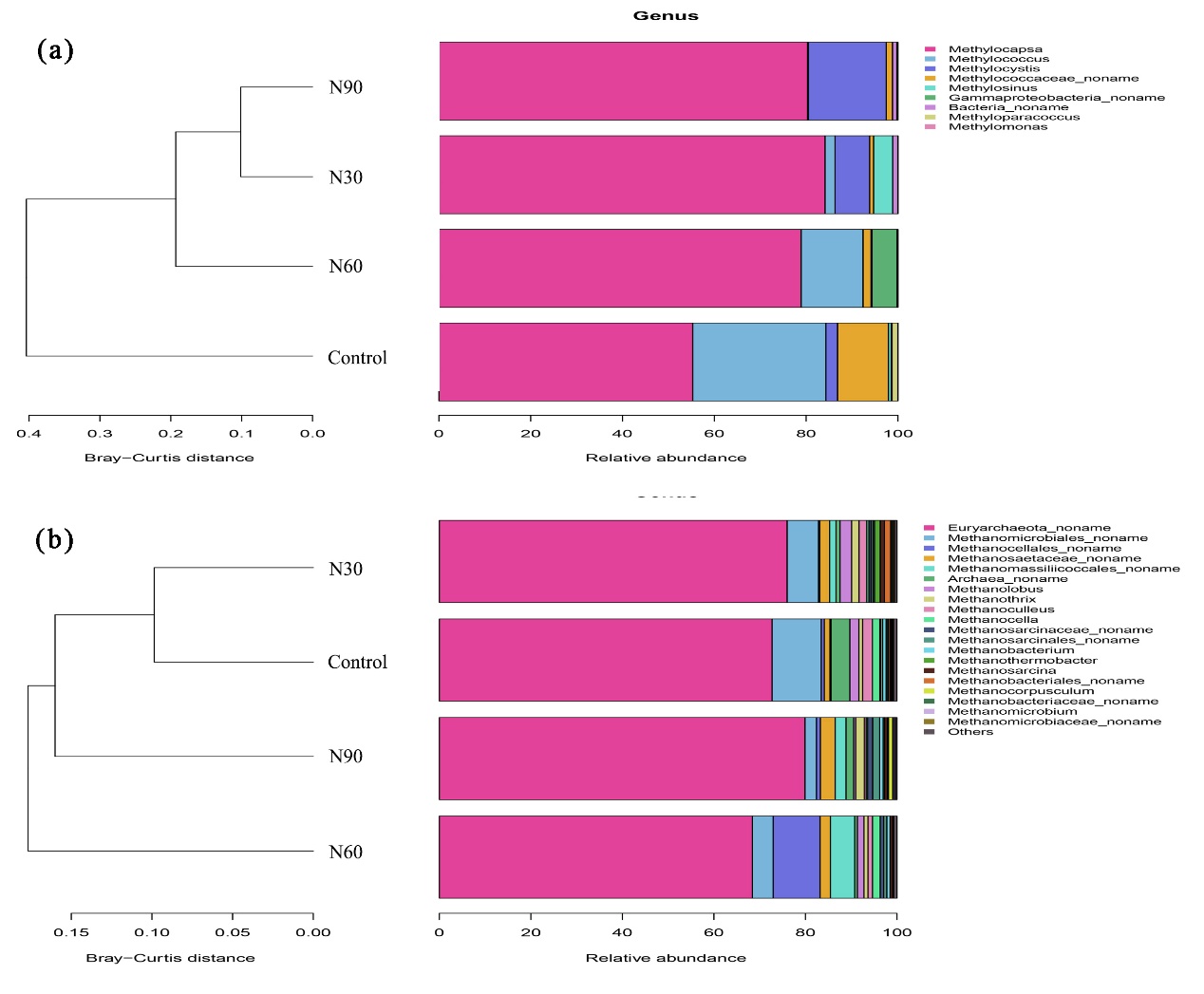
**

**Fig. S2**
